# A quest for stakeholder synchronization in the CAR T-cell therapy supply chain

**DOI:** 10.3389/fbioe.2024.1413688

**Published:** 2024-08-08

**Authors:** Shelby M. Holland, Amrik Sohal, Alka Ashwini Nand, Dietmar W. Hutmacher

**Affiliations:** ^1^ Department of Management, Monash Business School, Monash University Caufield Campus, Melbourne, VIC, Australia; ^2^ Australian Research Council Training Centre for Cell and Tissue Engineering Technologies, Monash University Clayton Campus, Melbourne, VIC, Australia; ^3^ Faculty of Engineering, School of Mechanical Medical and Process Engineering, Queensland University of Technology, Brisbane, QLD, Australia; ^4^ Australian Research Council Training Centre for Multiscale 3D Imaging, Modelling and Manufacturing (M3D Innovation), Queensland University of Technology, Kelvin Grove, QLD, Australia; ^5^ Max Planck Queensland Centre, Queensland University of Technology, Brisbane, QLD, Australia

**Keywords:** car-t, supply chain, logistics, collaboration, cell therapy, stakeholder coordination, cold-chain management

## Abstract

Advancements in cell therapy have the potential to improve healthcare accessibility for eligible patients. However, there are still challenges in scaling production and reducing costs. These challenges involve various stakeholders such as the manufacturing facility, third-party logistics (3PL) company, and medical center. Proposed solutions tend to focus on individual companies rather than addressing the interconnectedness of the supply chain’s challenges. The challenges can be categorized as barriers from product characteristics, regulatory requirements, or lagging infrastructure. Each barrier affects multiple stakeholders, especially during a boundary event like product handover. Therefore, solutions that only consider the objectives of one stakeholder fail to address underlying problems. This review examines the interconnecting cell therapy supply chain challenges and how they affect the multiple stakeholders involved. The authors consider whether proposed solutions impact individual stakeholders or the entire supply chain and discuss the benefits of stakeholder coordination-focused solutions such as integrated technologies and information tracking. The review highlights how coordination efforts allow for the implementation of widely-supported cell therapy supply solutions such as decentralized manufacturing through stakeholder collaboration.

## 1 Introduction

Advances in gene editing have paved the way for the development of cell therapies to treat cancers and inflammatory chronic conditions through extraction, modification, and re-infusion of patient cells. Some prominent lines of therapy include genetically modified T-cells to treat blood cancers, limbic stem cell modification to repair corneal epithelial cells, and allogeneic adipose stem cells to treat Chron’s disease (Bashor et al., 2022). In 2017, the Food and Drug Administration (FDA) approved chimeric antigen receptor (CAR) T-cell therapies tisagenlecleucel (labelled as Kymriah) and axicabtagene ciloleucel (labelled as Yescarta) for market use, prompting a surge in commercial development of the cell therapy industry (Bashor et al., 2022). Since then, stakeholders throughout the cell therapy industry have pushed for improvements to the scale of production, cost of treatment and logistics strategy for CAR T-cell therapies (Harrison et al., 2019; Papathanasiou et al., 2020).

A retrospective analysis of the outcomes for patients across tisagenlecleucel (Kymriah) clinical trials demonstrated 83% remission, indicating that Kymriah was effective in restoring quality of life to patients (Waldman et al., 2020). Despite the success of CAR T-cell in treating patients, there is a gap between the increasing demand for treatment and access to the treatment. According to a review of the cost and access of CAR T-cell therapies, only 2,500 patients received treatment between the years 2016–2020, even though there are approximately 3,000 patients eligible for treatment every year (Geethakumari et al., 2021). CAR T-cells did not receive market approval by the FDA until 2017, with lower number of patients initially. Still, there persists a significant patient access gap, particularly in Latin American, African, and Asia-Pacific countries (Odstrcil et al., 2024). Dimensions that contribute to this gap include complex logistics, long supply chains, limited medical centers to administer treatment, and the high cost of treatment (Harrison et al., 2019; Wei Inng Lim et al., 2022). Both developed and non-developed regions face these challenges, despite the different characteristics of the medical supply chains in each region (Odstrcil et al., 2024).

**FIGURE 1:**
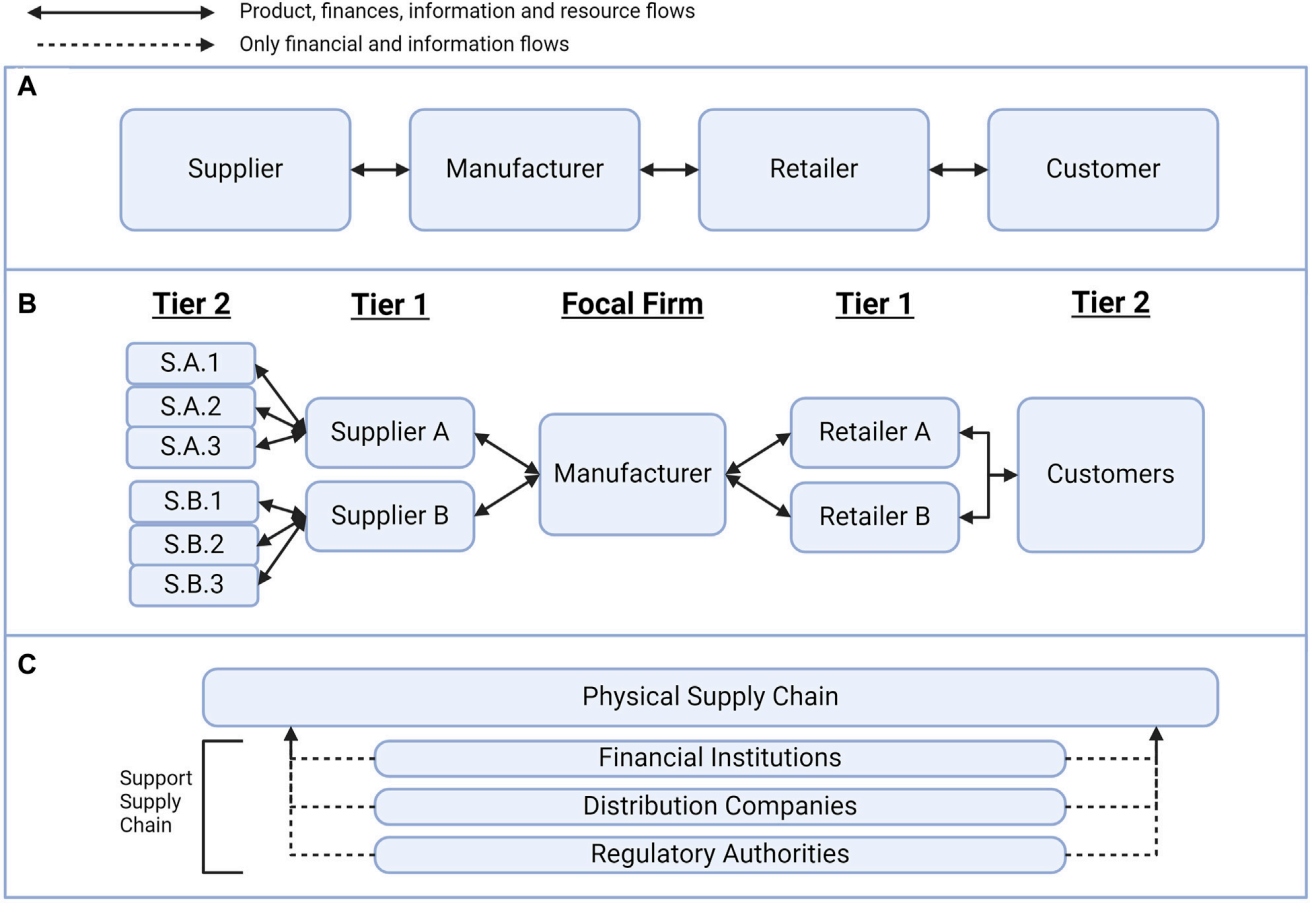
Supply chain management concepts. **(A)** The standard supply chain is the flow of resources, information and finances between stakeholders. **(B)** There are multiple tiers of suppliers, and the tier depends on the separation from the “focal firm” of a supply chain management strategy. **(C)** The support supply chain provides information and financial value to the physical supply chain without directly interacting with the product.

In response to this access gap, research in the logistics of CAR T-cell therapy aims to identify and mitigate some of the challenges of manufacturing, distributing and coordinating the global CAR T-cell supply chain (Lopes et al., 2020; Papathanasiou et al., 2020). As shown in Figure 1A, standard supply chains feature the flow of materials, resources, finances and information between stakeholders with a shared goal to bring value to the end customer (Chopra and Meindl, 2014). Supply chain maps can aid in visualising the multiple tiers of stakeholder involvement, which is determined by how far removed a supplier is from the “focal firm” for a management strategy, as shown in Figure 1B (Farris, 2010; Mubarik et al., 2021). Stakeholders in the physical supply chain often include raw materials suppliers, manufacturers, logistics companies and retailers as they directly provide value to the supply chain. Support stakeholders, as shown in Figure 1C, include financial institutions, advocacy groups, and accreditation or regulatory bodies as these activities provide value indirectly (Carter et al., 2015).

In the context of cell therapies, the patient serves as both the supplier and recipient of the treatment. As such, the relationships between physical and support stakeholders, as well as the various tiers of the supply chain, are highly interconnected (Lopes et al., 2020). As a result, further research analyzing stakeholder involvement in these cell therapies is needed to understand why the barriers to increased access are difficult to overcome. In this paper, the authors review the strategies proposed for improving the coordination of the CAR T-cell therapy supply chain. They also identify the challenges faced by manufacturing, logistics, and clinical stakeholders in the cell therapy industry. Furthermore, the paper discusses the impact of the interfaces between stakeholders on the ability to prioritize supply chain goals, implement proposed strategies, and overcome barriers to treatment access.

## 2 CAR T-Cell therapy

Chimeric antigen receptor (CAR) T-cells are genetically modified immune cells that specifically target hematological cancers such as leukemia and lymphomas (Beyer and Schultze, 2006; Hay and Cheung, 2019; Waldman et al., 2020). Kymriah (tisagenlecleucel) is a type of CAR T-cell therapy that is manufactured by pharmaceutical company Novartis, to treat adult patients with B cell precursor acute lymphoblastic leukemia (B-ALL) and diffuse large B cell lymphoma (DLBCL) that is refractory, which means that the patient was not responsive to previous treatments for B-ALL or DLBCL (Tyagarajan et al., 2020). There are other market approved CAR T-cell therapies including Yescarta (Axicabtagene ciloleucel), Tecartus (Brexucabtagene autoleucel), Abecma (Idecabtagene vicleucel), Carvykti (Ciltacabtagene autoleucel) and Breyanzi (Lisocabtagene maraleucel) which target a range of patients with refractory or relapsed blood cancers (Calmels et al., 2018; Bashor et al., 2022; Myles and Church, 2022; Velickovic and Rasko, 2022; Khan et al., 2024). Table 1 details some of the approvals for major markets, including Australia, the United States, Europe, and Japan (Novartis, 2020; Rodrigues et al., 2021; Johnson, 2022a; Johnson, 2022b; Malik et al., 2022; Gilead, 2023; Bristol Myers Squibb, 2024; Conway, 2024). Currently, all CAR T-cell therapies approved for market use are autologous therapies.

**TABLE 1:** FDA Approved CAR T-cell Therapies.

Name	Manufacturer	Global approvals	Price (USD)	FDA approval date
Kymriah (Tisagenlecleucel)	Novartis	26 countries (incl. US, AUS, JPN, EU)	$475,000	August 2017
			$437,927	May 2018
Yescarta (Axicabtagene ciloleucel)	Kite pharma	20 countries (incl. US, AUS, JPN, EU)	$375,000	October 2017
			$375,000	March 2021
Breyanzi (Lisocabtagene maraleucel)	Bristol-myers squibb	USA, JPN, EU, SWI, CAN	$470,940	February 2021
Tecartus (Brexucabtagene autoleucel)	Kite pharma	USA, AUS, EU	$373,000	July 2020
Abecma (Idecabtagene vicleucel)	Bristol-myers squibb and bluebird bio	USA, EU	$441,743	March 2021
Carvykti (Ciltacabtagene autoleucel)	Janssen biotech (Johnson & Johnson)	USA, AUS, EU	$465,000	February 2022

There are two main types of cell therapy modalities: autologous and allogeneic. The key difference between the modalities is whether the modified cells are extracted from the patient or from a cell donor. Allogeneic therapy involves modifying cells that are stored in a cell bank but are still in clinical development due to lower efficacy and the risk of graft versus host disease (Harrison et al., 2019; Geethakumari et al., 2021). One of the key benefits of allogeneic modalities is the simple supply chain which resembles a more traditional pharmaceutical supply chain (Lakelin, 2017). Allogeneic approval will disrupt the CAR T-cell market because of the improved responsiveness to demand fluctuations and subsequent reduced wait times for stakeholders downstream from manufacturers in the supply chain (Papathanasiou et al., 2020). However, only autologous modalities have received market approval.

**FIGURE 2:**
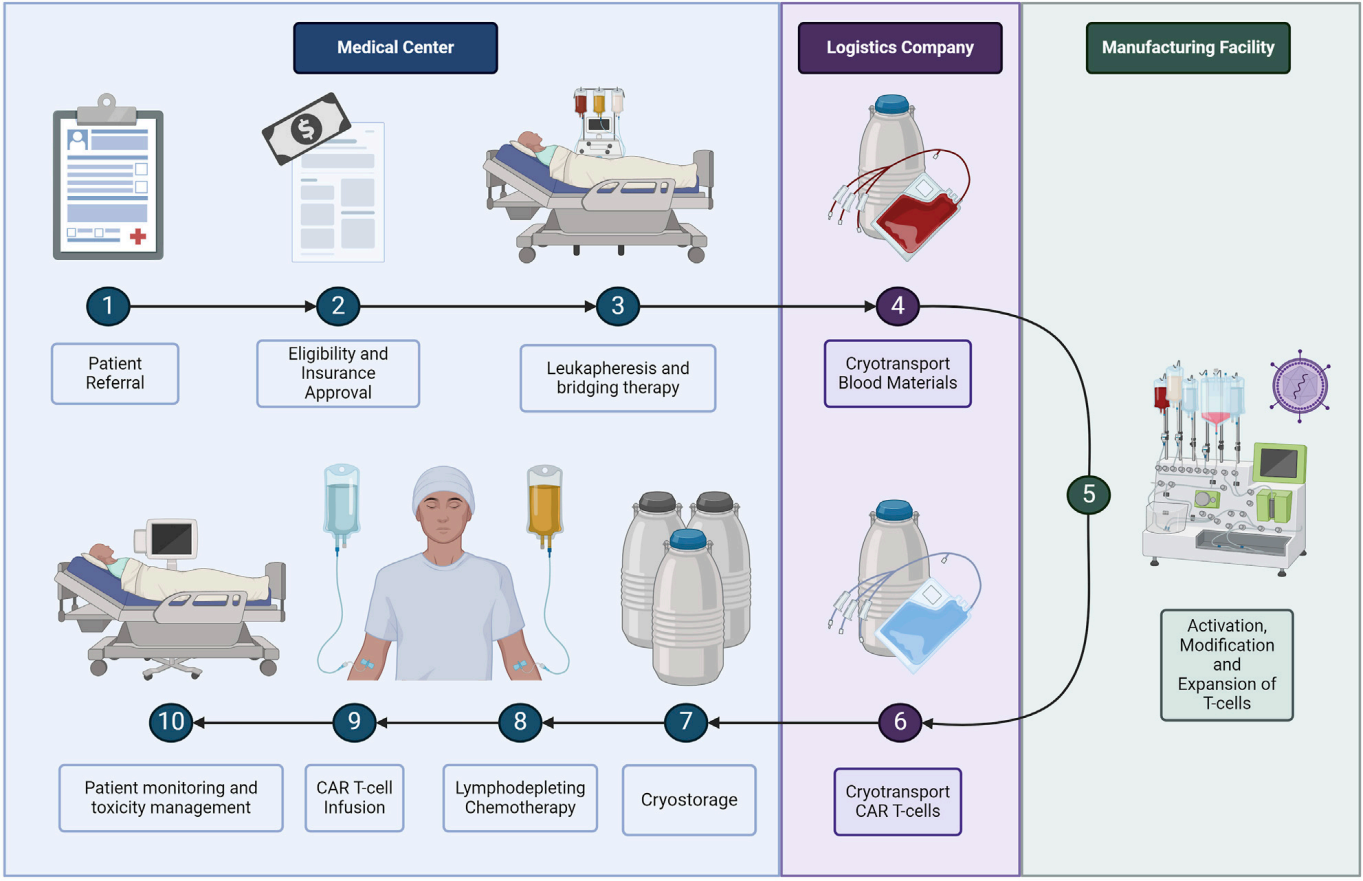
The CAR T-cell process through the physical supply chain stakeholders.

Although autologous CAR T-cell treatment reduces the risk of graft versus host and has a higher efficacy, the logistics involved are complex and expensive. The autologous CAR T-cell process starts when a patient is referred to a specialist clinic for evaluation and to initiate the approval process for reimbursement, as shown in Figure 2 (Buie, 2021). Once the patient is approved for treatment, the patient must return to the clinic to undergo leukapheresis, which is the extraction of blood products (Griffiths and Lakelin, 2017; Lopes et al., 2020). If the raw materials must be transported a long distance, or there is a longer expected wait time for manufacturing, the clinic will place the cells in cryogenic storage using liquid nitrogen (Harrison et al., 2019; Lopes et al., 2020). During transport, temperature and pH levels must be maintained and physical stress minimised to prevent cell loss (Li et al., 2019). At the manufacturing facility, the blood products undergo several processing steps including cell activation, CAR transduction using a viral vector for gene modification, and cell expansion (Kaiser et al., 2015). Next, the manufacturing facility will place the cells in transport tanks designed for cryogenic storage before the third-party logistics company transports the cells back to the treatment facility (Li et al., 2019; Karakostas et al., 2020; Lopes et al., 2020). The cryogenic tanks will wait in storage at the medical centre as the patient undergoes a week-long conditioning step before finally receiving the CAR T-cell therapy treatment (Li et al., 2019; Lopes et al., 2020). Treatment is followed by 4 weeks of monitoring in case of neurotoxicity side effects (Calmels et al., 2018; Borgert, 2021; Myers et al., 2021).

**FIGURE 3:**
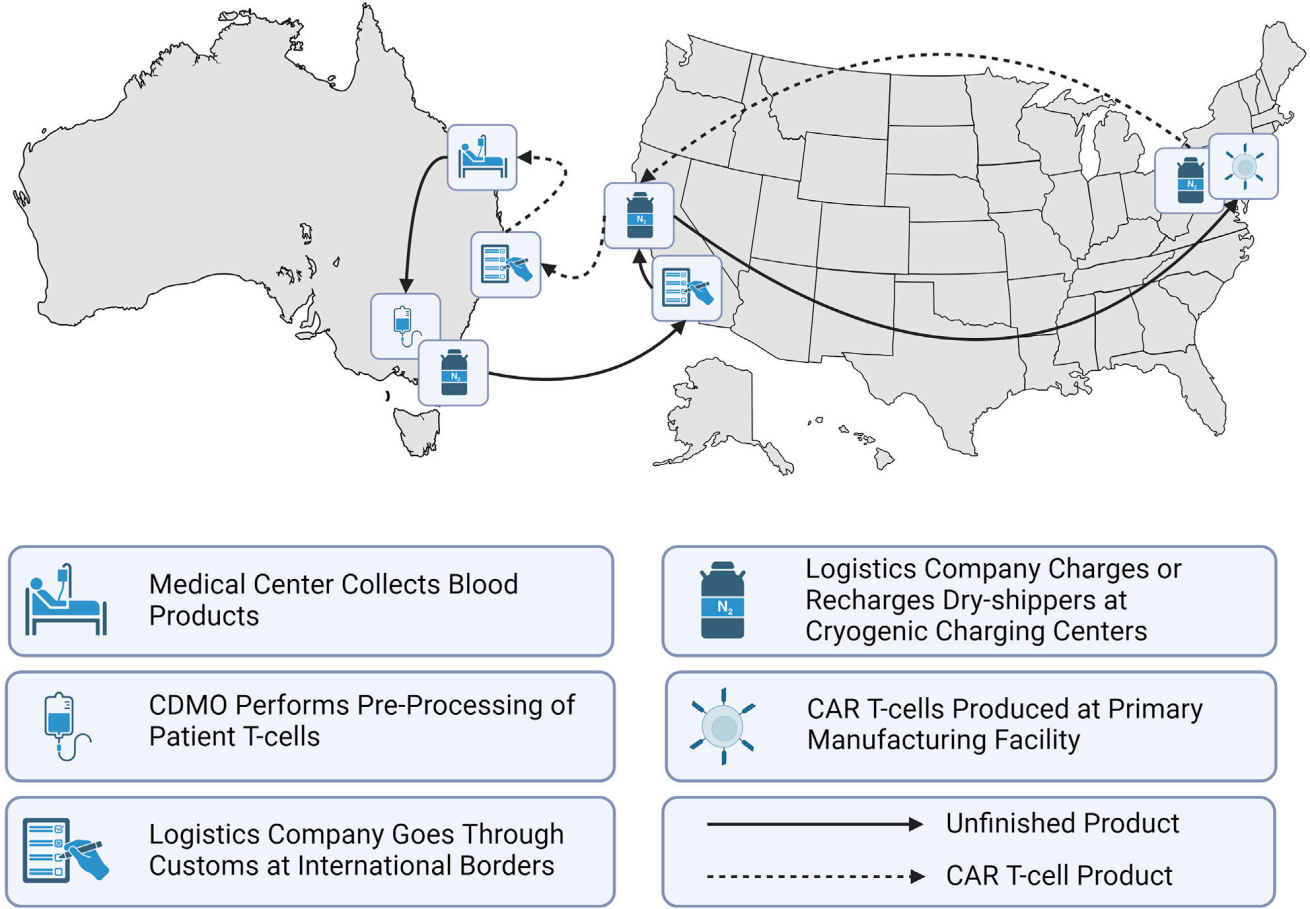
Representation of Australia’s CAR T-cell therapy supply chain when manufacturing occurs off-shore.

CAR T-cells were first approved by the United States Food and Drug Administration (FDA) in 2017 for medical use (Waldman et al., 2020; Bashor et al., 2022). Since then, the FDA has approved a total of six CAR T-cell therapies as shown in Table 1 with indications for both adult and paediatric patients to treat lymphoma, myeloma and leukemia (Khan et al., 2024). Regulatory bodies such as the FDA in the US or the Therapeutic Goods Administration (TGA) in Australia manage both market approvals that allow for the sale of biologics such as cell therapies and the manufacturing approval for facilities to safely produce the therapeutic (Velickovic and Rasko, 2022). Manufacturing approval has a significant impact on the supply chain topography for a given geographic region. Prior to manufacturing approval in Australia, the supply chain for Kymriah required extraction and intermediate materials processing and cryogenic freezing in certified facilities, transport across international borders to New Jersey (USA) for manufacturing, followed by a return to Australia as depicted in Figure 3 (Velickovic and Rasko, 2022). These lengthy transport times and border crossings introduce numerous risks due to customs clearance requirements, risk of temperature excursion events and errors in cell tracking. Manufacturing approval in 2021 for Cell Therapies in Melbourne, Australia means that an alternative stream for Kymriah is now available, where the cell extraction, manufacturing and treatment infusion occur without the need for international border crossings (Smith, 2023).

The cost of the CAR T-cell therapy is priced between US$300,000 – US$500,000 (Table 1), not including overhead costs during in-patient treatment, neurotoxicity treatments, transportation, and temporary housing for the patient (Lopes et al., 2020; Borgert, 2021; Palani et al., 2023). With these ancillary costs combined, treatment with CAR T-cell therapy can reach up to US$1 million for a patient (Borgert, 2021; Jenei et al., 2021). Therefore, reimbursement is a crucial aspect of treatment access for patients. The first model for reimbursement was based on the efficacy of treatment, where Novartis pledged a refund to patients who were not responsive to Kymriah (Boyiadzis et al., 2018). In the US, CAR T-cell treatments were covered for some patients by Medicare through bundled diagnosis-related group (DRG) codes for autologous bone marrow transplants. As there was a significant gap in costs between bone marrow transplant and CAR T-cell therapy, an additional New Technology Add-on Payment (NTAP) was added. Since then, CAR T-cell therapy has been designated its own DRG code (018) with improved coverage (Borgert, 2021). In countries like Australia, patients in certain population groups, including children and adults with acute lymphoblastic leukemia (ALL), are completely reimbursed through the Medicare Benefits Scheme (MBS) sponsored by the federal government (Hunt, 2020). Despite efforts to form reimbursement policy to meet patient needs, the current amount of reimbursement falls short of the actual costs of the therapy depending on factors such as patient travel, risk of toxicity events and treatment setting. As such, policymakers for both public and private insurance have a significant impact on the affordability of CAR T-cell therapy.

**FIGURE 4:**
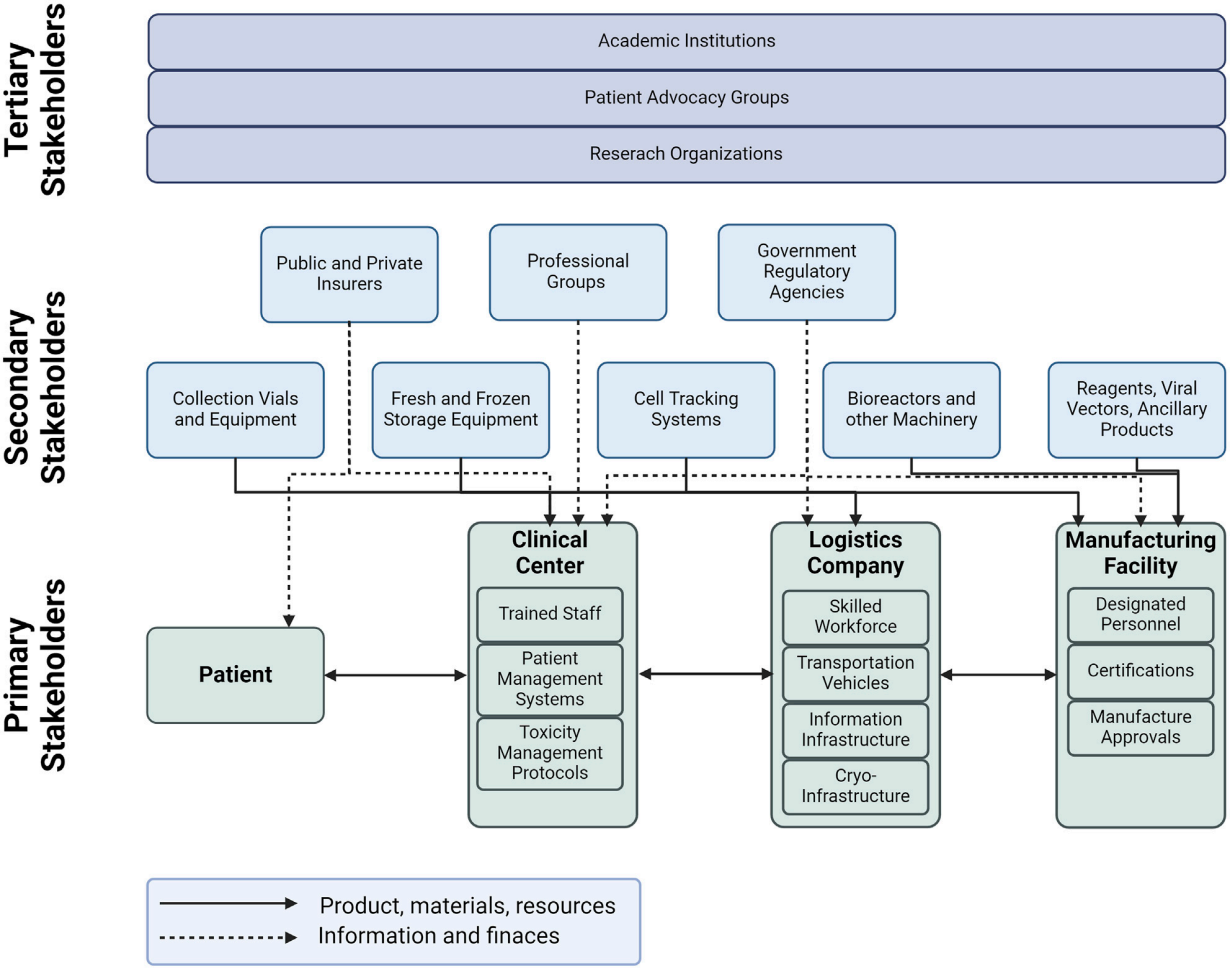
CAR T-Cell supply chain including the primary, secondary and tertiary stakeholders.

The cell therapy supply chain is a complex system that involves various stakeholders, divided into three types as shown in Figure 4. The first type includes the primary stakeholders who are directly involved in the value-adding activities for cell therapy. These stakeholders include the medical center, third-party logistics provider, and the manufacturing facility. The medical center is responsible for providing the necessary infrastructure for cell therapy, including the necessary facilities and trained personnel. The third-party logistics provider is responsible for the timely and safe delivery of cell therapy products to the patients. The manufacturing facility is responsible for the production of cell therapy products. The second type of stakeholders includes those who provide materials and other services to the primary stakeholders. These stakeholders provide raw materials, ancillary blood products, collection vials, equipment, training for personnel, and integrating technology for cell track and trace. The second type also includes support stakeholders such as government agencies, regulatory authorities, professional groups and insurance companies. Finally, the third type of stakeholders includes those who are indirectly involved in the cell therapy supply chain. These stakeholders include patient advocacy groups, academic institutions, and research organizations. Patient advocacy groups play a critical role in raising awareness about cell therapy and advocating for patients’ rights. Academic institutions and research organizations are involved in research and development of new cell therapy products and technologies.

The efficient functioning of the supply chain in the context of cell therapy highly depends on the seamless coordination and interaction among its different tiers. This interaction is vital to ensure that the patient receives a safe and effective product. However, most of the existing literature on the cell therapy supply chain has a narrow focus, analyzing the challenges faced by primary stakeholders in isolation. In this context, we aim to present a review that highlights the importance of considering the degree of interaction between all stakeholders, including secondary and support stakeholders, in order to gain a comprehensive understanding of the barriers that limit access to CAR T-cell therapy. This approach will help in developing effective solutions that will benefit all stakeholders involved in the supply chain and ultimately, improve patient outcomes.

## 3 Challenges of the CAR T-Cell supply chain

A thematic literature review was conducted by the authors to identify the challenges faced by the primary supply chain members. The review revealed that these challenges can be broadly categorized into three categories, namely product characteristics, and regulatory or infrastructure barriers. Product characteristic challenges relate to the requirements and specifications for the quality, safety, and efficacy of the product. These challenges may include, but are not limited to, the need for specialized equipment, materials, or training to meet the desired product specifications. Regulatory challenges involve the processes, certifications, and general requirements that stakeholders must meet to administer, manufacture, or transport the product. These challenges may include obtaining regulatory approvals, meeting industry standards, and complying with various safety regulations. Additionally, the associated costs of meeting these requirements can be significant, particularly for smaller stakeholders or those with limited resources. Infrastructure challenges include factors such as reimbursement, capital requirements, transportation routes, and skilled labour requirements that limit scaling for each stakeholder. These challenges may extend beyond the primary supply chain members to include other stakeholders such as logistics providers, distributors, and retailers. Inadequate infrastructure can lead to delays, inefficiencies, and increased costs across the entire supply chain. Overall, the challenges faced by primary supply chain members are multifaceted and can be complex to address. Identifying and understanding these challenges is the first step in developing effective solutions and improving the overall efficiency and effectiveness of the supply chain.

### 3.1 Clinical challenges for CAR T-cell therapy

The medical center is the starting point and ending point for the cell therapy supply chain. Within the medical center, patients undergo initial evaluation for treatment, go through the insurance approval process, provide the raw blood materials during leukapheresis, endure the conditioning step and receive the final treatment (Jenei et al., 2021). The medical center is the key interface between the patient and supply chain and therefore the challenges that the medical center contends will have impacts throughout the supply chain.

#### 3.1.1 Clinical challenges due to product characteristics

In spite of the improvement to patient’s quality of life, CAR T-cell therapy has risk of critical health side effects such as cytokine release syndrome (CRS) and immune effector cell-associated neurotoxicity syndrome (ICANS) (Calmels et al., 2018). These adverse side effects arise because of increased cytokine production associated with cell expansion in the case of CRS and T-cell activation in the case of ICANS, both of which are important mechanisms for CAR T-cell efficacy and persistence (Boyiadzis et al., 2018). CRS can show symptoms of fever and hypotension 2–5 days post infusion, and can lead to critical conditions such as cardiomyopathy, renal failure, pleural effusions and coagulopathy (Borgert, 2021). ICANS leads to activation of infused T cells typically between 4–10 days post infusion and can lead to encephalopathy, aphasia, seizures, and cerebral edema (Ortiz de Landazuri et al., 2020; Borgert, 2021; Myers et al., 2021). In-patient treatment of toxic events contributes to costs exceeding approved reimbursement amounts (Borgert, 2021; Buie, 2021). Due to the severity of this risk, patients are required to remain within 30 min of travel time of the medical center. This often leads to patients paying for extended stays in accommodation nearby medical centers for treatment preceding and following CAR T-cell infusion (Borgert, 2021; Buie, 2021; Sarkis et al., 2021).

Another challenge for medical centers is the state of health of the patient. Patients must be approved for treatment and in some cases approved for reimbursement. During this time, clinicians have reported a discontinuation of treatment to as many as 40% of patients because of health deterioration (Wayment, 2022). Patients must have sufficient healthy cells to start treatment, therefore deterioration prevents cell harvesting and in some cases infusion of the final product (Geethakumari et al., 2021).

While the product characteristics challenges of patient deterioration and toxicity events are primarily managed by the medical center, these issues impact the entire supply chain. The safety profile of a line of therapy is directed by developers such as the pharmaceutical company, while the financial impacts of toxic events are shaped by reimbursement policy for inpatient versus outpatient treatment. Reimbursement policy also impacts whether patient deterioration factors into the quality of the cells for the manufacturer. Overall, these challenges are interconnected with other stakeholders in the supply chain.

#### 3.1.2 Clinical challenges due to regulatory requirements

There are a disparate number of medical centers eligible to administer CAR T-cell therapy, which limits patient access. CAR T-cell administering medical centers require approval by the local regulatory body to administer therapy, which involves compliance with standards outlined by professional organizations. Thus, local regulatory bodies and professional organizations are part of the support supply chain and provide value through quality and safety assurance (Geethakumari et al., 2021).

There are several professional organizations that coordinate to establish robust standards for immune effector cellular therapy (IEC), including CAR T-cells. These organisations include the American Society of Hematology, American society for Blood and Marrow Transplantation (ASBMT), American Society of Gene and Cell Therapy (ASGCT), International Society for Cellular Therapy (ISCT) and the Foundation for the Accreditation of Cell Therapy (FACT) (London, 2018). Medical centers administering CAR T-cell therapy to patients worldwide require either FACT accreditation or Joint Accreditation Committee ISCT-Euromedical & EMBT accreditation (Wei Inng Lim et al., 2022). Accreditation indicates that the medical center is compliant with the standards established to ensure that sites provide high quality cell collection, cell processing and administration of hematopoietic (including CAR T-cell) therapy (Wei Inng Lim et al., 2022).

The standards formalized by FACT cover clinical programs, the collection of cell materials, and cellular processing since some hospitals may be linked to academic institutions that engage in manufacturing steps (Geethakumari et al., 2021). These standards have been formalized as the Standards and Accreditation Program as of 2017, which involved collaboration between FACT, ISCT, ASGCT and the Society for Immunotherapy of Cancer (SITC) as well as experts in academia. Every 3 years, FACT requires an audit of accredited facilities that covers:

1) The location of cell manufacturing unless manufactured at a third-party site.
2) Adverse event management including cytokine release syndrome (CRS) and neurotoxicity events.
3) Coordination and communication of clinical staff, including the ongoing training and education of providers involved in CAR T-cell administration.
4) Data reporting, which involves product safety and outcomes for future review of product safety and efficacy profiles.

These standards are guided by the Risk Evaluation and Mitigation Strategies (REMS) programs developed by the FDA to guide adverse event management. The REMS system also determines how frequently and to what extend providers are trained in identifying CRS and neurotoxicity events post-infusion with CAR T-cell therapy (Buie, 2021; Geethakumari et al., 2021).

Outside of the United States, cell therapies have additional regulatory requirements depending on the country and are often designated as advanced therapy medicinal products (ATMP). This designation requires additional oversight by the relevant regulatory bodies such as the European Medicines Agency (EMA) and the Therapeutic Goods Administration (TGA) in Australia (Geethakumari et al., 2021).

Pharmaceutical companies such as Novartis, also require medical centers to demonstrate accreditation to administer their cell therapy product (Tyagarajan et al., 2019). When Kymriah was granted market approval by the TGA in December of 2018 in Australia, the first medical center Novartis approved to administer the therapeutic was the Royal Prince Albert Hospital (RPAH) in Sydney, NSW (Velickovic and Rasko, 2022). In 2019, Novartis conducted an audit including good manufacturing practices (GMP) standards according to Therapeutic Goods Order 88 and the Code of Good Manufacturing Practice for Human Blood and Blood Components, Human Tissues, and Human Cellular Therapy Products (Velickovic and Rasko, 2022). The formalized agreement with Novartis was in the form of a Technical Apheresis Agreement with the Sydney Local Health District, which allowed RPAH to be the first accredited site to administer Kymriah (Velickovic and Rasko, 2022). Since then, RPAH and other approved medical centers to administer CAR T-cells have obtained FACT accreditation.

Oversight by accreditation organizations and regulatory bodies and involvement of the primary manufacturer (i.e. the pharmaceutical company) demonstrates that multiple stakeholders in the supply chain prioritize patient safety at each stage of the process. However, the burden of cost, training, resource management and ultimately the risk of toxicity events still rests on the clinical center. As a result, it is difficult to expand the number of medical centers capable of administering CAR T-cell therapy, which would expand access to care for patients worldwide. Professional organizations outlined the standards to improve patient care in the event of toxicity through a collaborative process that involved multiple stakeholders. Novartis worked closely with RPAH to ensure that appropriate protocols were in place to ensure patient safety, because of the mutual interest to bring Kymriah to the Australian market. In order to further expand the reach of CAR T-cell therapy worldwide, there must be similar support to reduce the financial and operational burdens of medical centers seeking to gain certification.

#### 3.1.3 Clinical challenges due to infrastructure

The sparse number of CAR T-cell medical centers leads to a bottleneck in hospital capacity to store and deliver treatment to a growing population of eligible patients. The containers used to transport the final product are large and bulky as they are designed to store cells cryogenically. Hospitals are constrained by the amount of space they have for a very specific storage requirement Medical centers do not typically have a storage space purpose-built for such a niche patient population group, and so space-constrained hospitals struggle with long-term storage of the CAR T-cell product (Karakostas et al., 2020; Ortiz de Landazuri et al., 2020). Additionally, hospitals require specialized infrastructure for receiving, storing and administering the treatment (Lopes et al., 2020). One solution proposed has been the use of mobile medical units that travel to the patient for product infusion (Karakostas et al., 2020). This solution would target the space optimization problem in hospitals and reduce the cost burden of patients travelling. However, this solution has a high risk of transferring other costs to patients due to the regulatory requirements for treatment centers and may have challenges in navigating the reimbursement space.

Reimbursement is a significant portion of infrastructure for CAR T-cell therapy in the healthcare space. The schemes for recovering costs for hospitals and patients alike have evolved since the initial approval of Kymriah in 2017 by the FDA. The process for insurance approval in the United States can take up to 30 days and is required prior to the cell-collecting apheresis step (Geethakumari et al., 2021). Patients pursue CAR T-cell therapy as a last line of treatment, so the risk of their deterioration while waiting for insurance approval is significant and has led to many patients falling out of eligibility (Wayment, 2022).

Even when patients are granted reimbursement for their treatment, the total cost may exceed coverage for a variety of reasons including toxicity events requiring intensive care, hospital administration costs, and other on-costs associated with in-patient treatment (Davies and Rafiq, 2017; Harrison et al., 2019; Ran et al., 2020; Borgert, 2021). Inpatient costs related to managing toxicity events accounted for 37.5% of the total cost of treatment according to an economic model developed for treatment with Kymriah in the US (Yang et al., 2020; Myers et al., 2021). Thus, reducing the risk of toxicity directly impacts the amount of coverage patients receive for the cost of care simply by shifting from out-patient treatment to in-patient treatment.

Outpatient treatment has been demonstrated to minimize costs and provide more optimal reimbursement outcomes for patients (Borgert, 2021; Buie, 2021; Geethakumari et al., 2021; Borogovac et al., 2022). Medicare pays for CAR T-cell in outpatient settings through patient co-pay based on daily services, which is capped at US$1,408, meaning fuller coverage for patients compared to inpatient treatment (Myers et al., 2021). Outpatient treatment relies on managing and minimizing toxicity related events and constant caregiver support for patients undergoing treatment (Borgert, 2021; Myers et al., 2021; Borogovac et al., 2022). A safety and feasibility study on outpatient care conducted at a tertiary care center maintained an overall admission rate of 24% within 72 h of treatment infusion using four different CAR-T products, and reported no emergency room visits or treatment related deaths (Borogovac et al., 2022). The study reported on the eight components of their outpatient treatment program (Borogovac et al., 2022). This included:

1) Maintenance of a multidisciplinary team.
2) Competent nursing staff.
3) Education of community providers.
4) Augmentation of patient knowledge.
5) Acquisition of physical space.
6) Adherence of policy and procedure.
7) Review of financial outcomes.
8) Continuous review of outcomes and procedures.

Establishing outpatient facilities with comprehensive toxicity management programs can lead to better reimbursement. However, this solution requires a substantial investment in clinical infrastructure, adherence to relevant procedures and policies, and consistent communication with patients to ensure compliance. Therefore, stakeholder collaboration is essential, involving joint investment in such programs to share financial risks as well as benefits.

### 3.2 Logistics challenges for CAR T-Cell therapy

One-quarter of the overall cost of commercializing a cell therapy product line is due to logistics (Myles and Church, 2022). The coordination between the treatment facility, patient, logistics company and manufacturing facility as well as the requirements around storage, quality, timing, and risk management means that the logistics are complex and expensive (Karakostas et al., 2020; Papathanasiou et al., 2020; Triantafyllou et al., 2022). Although cold-chain logistics is well established in the pharmaceutical industry, cell and gene therapies (CGTs) pose unique product challenges, regulatory requirements and infrastructure barriers that directly involve the logistics stakeholder. These unique needs of the cell therapy supply chain have led to a move towards third party logistics companies (3PLs) that provide logistics that prioritizes maintaining product quality throughout the supply chain.

#### 3.2.1 Logistics challenges due to product characteristics

CAR T-cell therapies are cell based, and hence are subject to the product limitations that biologics face. Cells are sensitive to temperature, pH and mechanical strain induced by vibrations and shear stress. Outside of specific temperature ranges, cells experience degradation and cell-death which reduces the amount of safe, high-quality product available for manufacturing (Li et al., 2019). The cells may exceed this temperature range during what is called a temperature excursion event, which can happen during handling steps in manufacturing as well as handover steps during transportation between facilities (Griffiths and Lakelin, 2017; Li et al., 2019). Additionally, cells have delicate structures that can be compromised by vibrations from handling and transport, for example in a truck, which leads to damaged cells and loss of product (Sarkis et al., 2021).

Cells can be transported fresh or cryopreserved depending on how long the cells need to remain in storage. Typically, if cells must be transported over long distances for manufacturing or are waiting for the patient to undergo lymphodepletion ahead of treatment, the best standard is to store the cells cryogenically. Cell storage with liquid nitrogen maintains a temperature of −180°C, which extends the product’s shelf life thus providing flexibility in supply chain timelines (Calmels et al., 2018; Papathanasiou et al., 2020; Triantafyllou et al., 2022). Cryopreservation involves suspending the cells in a cryo-agent solution that is suitable for freezing and thawing, as the cycle between states can also lead to damaged cells. Another risk in the cryogenic storage process is cell loss from biochemical toxicity of the cryo-agents (Li et al., 2019). Cryogenic “dry-shippers” are specially designed to reduce tipping over and effects from vibrations that can lead to cell-damaging shear stress and require “re-charging” with liquid nitrogen for longer transportation routes along the supply chain (Griffiths and Lakelin, 2017). As such, cryopreserved transport is a more expensive form of delivering treatment across the supply chain. While cryo-shipping improves scheduling flexibility and coordination with other stakeholders, requirements around patient data security, chain of custody and hand-offs are complicated by this form of transportation (Griffiths and Lakelin, 2017; Calmels et al., 2018; Karakostas et al., 2020).

One of the unique scheduling challenges of this supply chain is the pre-conditioning step for the patient to go through lymphodepletion prior to CAR T-cell infusion (Borgert, 2021). The medical center and manufacturing facility must coordinate with each other to ensure that the product will be ready for infusion and the patient will be ready for treatment (Papathanasiou et al., 2020; Triantafyllou et al., 2022). The logistics company involved in cold-chain transport of the product must coordinate with both the manufacturer and the medical center for the hand-offs which involve checkpoints on chain of custody and cryogenic storage in the “dry-shipper” (Papathanasiou et al., 2020; Sarkis et al., 2021; Triantafyllou et al., 2022). The hospital must also store these large “dry-shippers” on site while the patient is undergoing the lymphodepletion step (Papathanasiou et al., 2020; Borgert, 2021). On a small scale, this is a manageable albeit expensive logistics problem.

Cloud-based information sharing has been proposed to improve coordination through information integration across the supply chain (Papathanasiou et al., 2020; Sarkis et al., 2022). However, information sharing is only one aspect of stakeholder coordination required to scale logistics capabilities within the constraints of CAR T-cell product characteristics. As more patients attempt to receive this treatment, this logistics problem becomes more unmanageable at the interfaces between the supply chain stakeholders.

#### 3.2.2 Logistics challenges due to regulatory requirements

Regulatory authorities require close monitoring and tracking of temperature excursion events and other quality control checks during transportation. This is completed as part of the chain of custody maintenance as the product is handed off between stakeholders in the supply chain (Branke et al., 2016; Papathanasiou et al., 2020; Sarkis et al., 2021). Not only is this required for quality control measures, but also as a way of maintaining patient data security, (Griffiths and Lakelin, 2017; Triantafyllou et al., 2022). The chain of custody requirement increases logistics complexity by adding extra check points and steps as well as additional data collectionto maintain. Track and trace technologies are a suggested solution to reduce logistics complexity and improve information integration between supply chain stakeholders while maintaining chain of custody of the product (Branke et al., 2016; Sarkis et al., 2022).

Another benefit of track and trace technologies is easing the complication that arises from variations in regulatory requirements for biologics logistics across international borders. There are restrictions on raw materials as well as intermediate material transfer across geographic boundaries that complicate the processes that cold-chain logistics companies must follow (Branke et al., 2016; Harrison et al., 2019). Additionally, variations in international customs laws means that any cell-based products crossing international boundaries must go through customs clearance, which introduces risks to product quality through temperature excursion events (Griffiths and Lakelin, 2017). The sale of blood products is also regulated differently based on jurisdiction, which impacts the sovereign capabilities of manufacturing facilities worldwide as well as the supply chain routes for the logistics company (Geethakumari et al., 2021).

The management of data handling, chain of custody, and cell quality during transport is primarily the responsibility of the logistics company. However, these processes have significant consequences throughout the entire supply chain. By coordinating among stakeholders, a comprehensive set of international standards can be established to ease the burden on logistics companies while ensuring that shared supply chain goals for quality and safety are met.

#### 3.2.3 Logistics challenges due to infrastructure

A key infrastructure challenge for logistics is the lack temporary storage of the cryopreserved product (Wayment, 2022). For longer supply chains where a 3PL company transports cells cryogenically, the cells may need to be re-charged with liquid nitrogen and specially built “cryo-charging” stations. These stations must be placed strategically on key supply chain routes so that the cells can reach the primary manufacturer in an appropriate time frame (Zobel, 2023).

Overall, the key infrastructure challenge that the CAR T-cell supply chain faces is the difficulty in creating an “off-the-shelf” supply chain. The structure of each CAR-T supply chain will depend on factors such as patient populations, existing transportation infrastructure, regulatory requirements, manufacturing capabilities and treatment center infrastructures (Davies and Rafiq, 2017; Griffiths and Lakelin, 2017). Depending on the intellectual property rights, involvement with government and academic institutions, and licensing rights of contract manufacturers, the stakeholders involved in the supply chain may vary (Calmels et al., 2018; Sparrow, 2021; Triantafyllou et al., 2022). Suggestions on how to design the cell therapy supply chain look to existing cold chains with similarities such as the blood and vaccine supply chains. This is because of the similarity in the quality control requirement, sensitivities to temperature, fluctuations in demand and involvement of patient, treatment center, manufacturing facility and logistics provider (London, 2018; Karakostas et al., 2020). However, key differences in the product characteristics such as the critical adverse effects, patient-related scheduling constraints and strict regulatory requirements that are enforced for biologics limits the extent to which the cell therapy supply chain can emulate these well-established cold chains. As such, “logistics-by-design” solutions that create a supply chain strategy through joint-development with clinical, manufacturing and logistics stakeholders is a more flexible, de-risked solution compared to “off-the-shelf” (Myles and Church, 2022).

### 3.3 Manufacturing challenges for CAR T-Cell therapy

Manufacturing involves the processing steps of the blood materials obtained from the patient, including cell activation, transduction with a viral vector for gene editing, and cell expansion (Calmels et al., 2018). Handling patient cells involves risks that range from temperature excursion events to batch failure due to a processing error (Saha, 2021). Considering how few cells are available for processing and the invasive, volatile nature of the injectable therapeutic, these risks can be catastrophic for the patient (Kaiser et al., 2015; Boyiadzis et al., 2018). As a result, there are strict regulatory requirements involved in the processing, handling and storage of raw materials needed for manufacture which require GMP certification to prove that appropriate controls are in place (Britten et al., 2021; Ravindranath et al., 2022). Thus, manufacturers, for example, Novartis who own the rights to commercialize Kymriah, must invest heavily in infrastructure, personnel, and regulatory approvals to safely produce the cell therapy.

Contract development and manufacturing organisations (CDMOs) contract with the proprietary owner of the patent for the therapeutic to produce the product in a facility with appropriate GMP certifications (Sorensen, 2023). CDMOs and academic institutions are a form of decentralized manufacturing, which can reduce the inventory burdens and logistics complexities for patients to allow better supply chain scaling (Karakostas et al., 2020; Wei Inng Lim et al., 2022). While there is a significant potential for scaling across the globe by utilizing CDMOs, the manufacturer faces non-trivial challenges as a supply chain stakeholder.

#### 3.3.1 Manufacturing challenges due to product characteristics

In general, cell therapy products have a high per-unit manufacturing cost (Harrison et al., 2019; Papathanasiou et al., 2020; Borgert, 2021). This is in part due to the high materials costs, which include the raw materials such as viral vectors and specialized reagents. Materials used in the production of CAR T-cell therapy are more expensive when they are chemically based, but have the advantage of less variability and risk of contamination which improves the safety profile of the end product (Harrison et al., 2019; Ran et al., 2020). These raw materials include antibodies for the activation and enrichment step, DNA plasmids, RNA and viral vectors for genetic modification, cytokines for the expansion medium, cell-washing for formulation and cryopreservation materials such as dimethyl sulfoxide (Li et al., 2019; Papathanasiou et al., 2020). As the cell therapy industry scales globally, competition by suppliers of reagents may drive down raw material costs and expand sovereign capabilities for manufacturing facilities across the globe (London, 2018; Harrison et al., 2019).

The starting patient material is one of the most critical product-related barriers to overcoming manufacturing challenges. CAR T-cell therapies are biologics, which means that the treatment is derived from live cells. The therapeutic is made by modifying the surface receptors of a patient’s T-cells, which means that the starting material will be unique for each batch. The number of T-cells that can be extracted from the patient depends on the age, weight, progression of the disease state and the health of the patient (Boyiadzis et al., 2018; Calmels et al., 2018; Sarkis et al., 2021). If the patient has deteriorated and cannot provide sufficient T-cells for manufacturing the product then they cannot go forward with the treatment. Also, patients who are eligible for CAR T-cell therapy usually were not responsive to previous forms of treatment and have reached an advanced stage of their disease progression. This means that the starting blood materials are difficult to obtain as patients have limited cells to spare (Branke et al., 2016; Calmels et al., 2018; Buie, 2021). As a result, volumetric scaling is not feasible. Thus, manufacturing facilities are not only dealing with variable batch sizes and limited starting material leading to limited volumes of product but the catastrophic levels of risk if there is a batch failure (London, 2018; Buie, 2021; Sarkis et al., 2021). Although isolated product streams can mitigate some of the difficulties in dealing with variable batch sizes when manufacturing for multiple patients at once, this remains an unresolved challenge (Harrison et al., 2019).

#### 3.3.2 Manufacturing challenges due to regulatory requirements

CAR T-cell therapies are biologics, which carry a significant regulatory burden in most jurisdictions. Unlike traditional pharmaceuticals, autologous CAR T-cells are formulated with a variable starting material so alterations to the manufacturing process may impact compliance with regulatory standards (Papathanasiou et al., 2020; Borgert, 2021; Sarkis et al., 2021). Thus, there is limited ability to alter any of the manufacturing processes to accommodate lower costs, efficient production, and volumetric scaling.

The major cost driver from regulatory compliance is the need for facilities to maintain GMP related certifications and perform quality audits (Boyiadzis et al., 2018; Calmels et al., 2018; Ran et al., 2020; Britten et al., 2021; Palani et al., 2023). Quality control (QC) assesses critical quality attributes (CQAs) by a designated qualified person (QP) who is authorized to release the formulation to the clinical site if the sample demonstrates sufficient safety and efficacy (Papathanasiou et al., 2020). Starting materials are also subject to QC testing such as testing the T-cells for bacterial or fungal contaminants or the potency of the vector to ensure sufficient patient cell transduction (Levine et al., 2017). These quality audits are time consuming, expensive and require skilled, designated personnel which restricts scheduling flexibility for staffing. While these regulatory challenges complicate the manufacturing process, ensuring that the product is safe and potent is valuable to all stakeholders in the supply chain.

While decentralized manufacturing is often proposed as a solution to the challenges surrounding the production of CAR T-cell therapy, establishing and maintaining GMP across a series of decentralized manufacturing centers requires a significant amount of investment (Harrison et al., 2019). Automation and outsourcing of quality control and quality assurance may reduce some of the difficulty in managing GMP in a decentralized manufacturing setup (Branke et al., 2016; Papathanasiou et al., 2020). However, there are still significant costs in establishing the specialized infrastructure needed for a decentralized manufacturing setup while maintaining a standardized process that can ensure product safety and efficacy. This particularly complicates efforts to establish small manufacturing facilities in academic institutions connected to a cell therapy administering clinical center (Ran et al., 2020). One of the key benefits of CDMOs is that a single manufacturing facility can maintain staffing levels, and GMP certifications, and provide temporary storage for raw materials because they cater to more than one line of therapy. This also means that CDMOs can de-risk demand fluctuations to a certain degree to prevent stock-outs of common reagents used in CAR T-cell therapy lines (Sorensen, 2023). As such, CDMOs are the halfway point between centralized manufacturing through the pharmaceutical company and expensive small-scale production in academic institutions.

#### 3.3.3 Manufacturing challenges due to infrastructure

Centralized manufacturing carries significant costs, in part because these facilities cannot maintain batch productions in continuous runs due to the high variability in demand and challenges with accurate demand forecasting (Papathanasiou et al., 2020; Sarkis et al., 2022). Demand variability also increases the risk of facility disruption since redundancies in both machinery and facility capabilities are difficult to justify (Griffiths and Lakelin, 2017; Karakostas et al., 2020; Ortiz de Landazuri et al., 2020). Stakeholder coordination can improve information integration to improve demand forecasting in both centralized and decentralized manufacturing setups (Melo et al., 2009; Haghjoo et al., 2020; Papathanasiou et al., 2020). For example, the medical center can communicate an expected influx of referrals for treatment to let the manufacturing facility estimate the number of raw materials to have in stock (Papathanasiou et al., 2020). This would make the supply chain more dynamic and flexible to adjust for demand fluctuations.

Another cost driver for manufacturing is the need for specialized, skilled labor (Harrison et al., 2019; Lopes et al., 2020; Ortiz de Landazuri et al., 2020; Papathanasiou et al., 2020; Triantafyllou et al., 2022). Automation using solutions such as the CliniMACs Prodigy and other closed loop bioreactors can alleviate some of the cost burden and aid in establishing decentralized manufacturing (Ran et al., 2020; Britten et al., 2021; Palani et al., 2023). However, there is a risk of increasing the manufacturing time when automating steps of the process such as T-cell expansion as this can create a capacity bottleneck (Lopes et al., 2020; Sarkis et al., 2021). Establishing intermediate storage may de-bottleneck production as well as mitigate some of the demand fluctuation issues (Sarkis et al., 2021; Triantafyllou et al., 2022).

## 4 Opportunities for collaboration in the cell therapy supply chain

The leading literature on supply chain management involving multiple stakeholders encourages the identification of common goals to promote supply chain coordination (Melo et al., 2009; Cao and Zhang, 2011; Talavera, 2013; Triantafyllou et al., 2022). Stakeholders in a single supply chain may have different overall objectives, such as a logistics company minimizing inventory holding time, while a medical center measures patient turnover rates as an indicator of success. However, when attempting to improve supply chain coordination, identifying the aligned goals allows stakeholders to orient their individual objectives towards a shared incentive (Chandra & Kumar, 2001; McLaren et al., 2002; Ferreira de Araújo Lima et al., 2020; Wijewickrama et al., 2021). First, shared problems must be identified to begin defining the shared goals of supply chain members.

**FIGURE 5:**
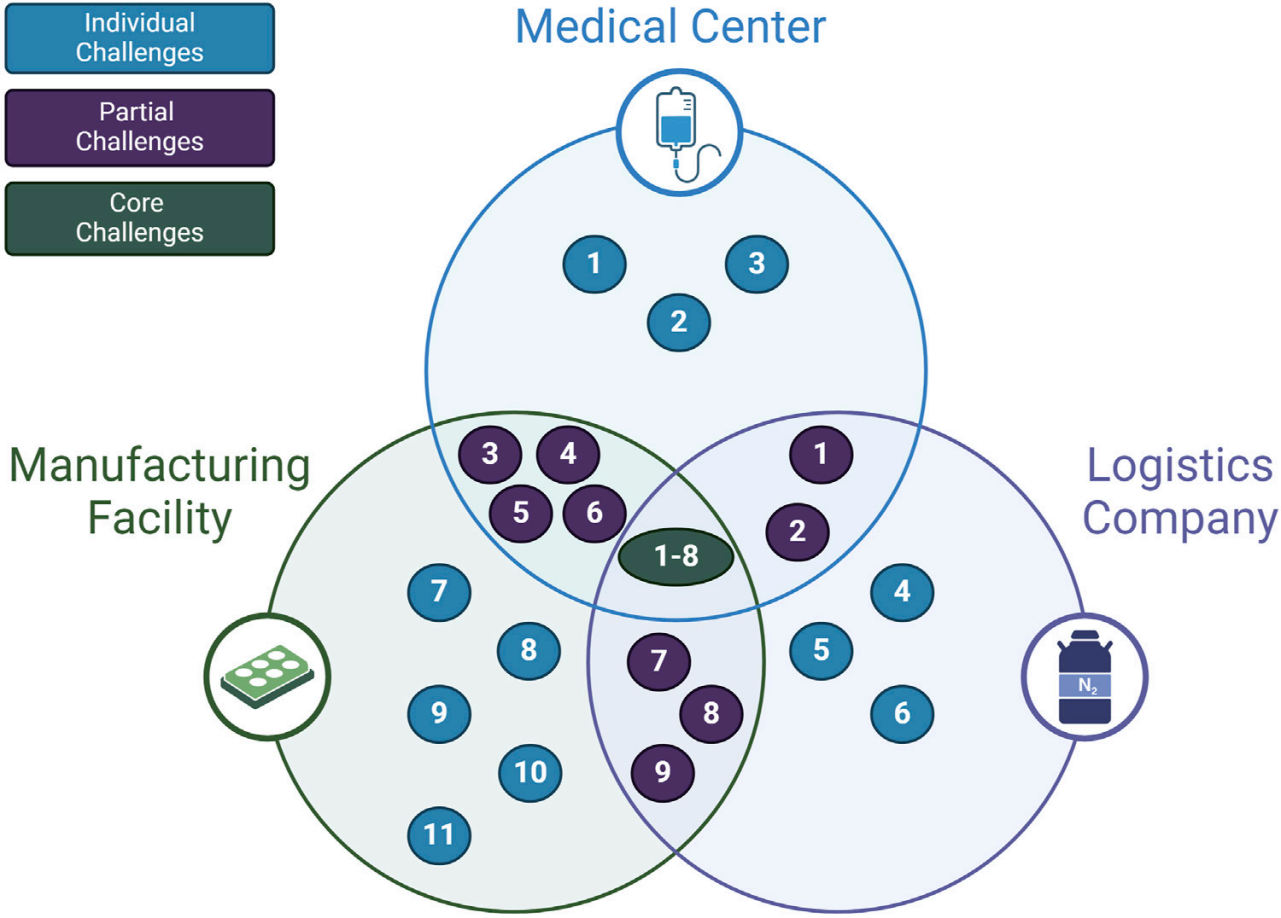
Mapping of stakeholder challenges shared by the primary physical supply chain.

So far, the presented challenges and opportunities of the cell therapy supply chain in this review are sequestered into separate categories. Another way of understanding the full scope of the challenges affecting the supply chain is to consider how stakeholders mutually experience these problems. As shown in Figure 5; Table 2, some problems affect all primary supply chain members. Requirements that preserve cell quality such as maintaining appropriate temperature, minimizing shear stress, and reducing cell loss during freeze-thaw steps are in the direct interest of the manufacturer, clinic, and logistics company (Harrison et al., 2019; Li et al., 2019). Thus, one shared goal for the supply chain may be the accurate tracking of quality measures for the cells. The desire to improve quality tracking has been identified by the literature in this review, but never positioned as a shared goal that can be achieved through collaboration between supply chain stakeholders.

**TABLE 2:** Shared challenges of the CAR T-cell supply chain, as shown in Figure 5.

Individual Challenges (1 Stakeholder)	Partial Challenges (2 Stakeholders)	Core Challenges (3 Stakeholders)
1. Administration centers require certification	1. Capacity bottlenecks at hospitals	1. Cells are sensitive to vibrations and shear stress
2. Risk of graft vs host for allogeneic therapy	2. Lack of infrastructure for temporary storage	2. Temperature excursions put cell quality at risk
3. Costs exceeding reimbursement thresholds	3. Starting material is difficult to obtain	3. Cells may be damaged from biotoxicity of cryo-agents
4. Too many variables to enable “off-the-shelf” SC	4. Variable batch sizes due to patients as source of materials	4. Demand forecasting is difficult to accurately predict
5. Regulatory restrictions vary by geography which complicates border crossings	5. Insurance approval required which causes delays and can lead to patient deterioration	5. Cryopreservation complicates security and coordination
6. Expensive and complex logistics	6. Patient deterioration restricts cell harvesting and infusion	6. Specialized infrastructure required
7. High materials costs for manufacturing	7. QC/QA and GMP makes decentralization difficult	7. Sample identification needed for chain of custody
8. Limited ability to alter manufacturing	8. Centralized manufacturing expensive	8. Pre-conditioning step for the patient requires scheduling
9. Risk of facility disruption and inaccurate demand forecasting	9. Toxicity management requires 3 weeks patient monitoring	
10. Skilled labor required		
11. T-cell proliferation step is a bottleneck in manufacturing		

Partial challenges that appear to apply to two interacting stakeholders are also vital for aligning supply chain goals. For example, the problem that starting material is difficult to obtain and patient deterioration due to insurance approval delays is a challenge that directly affects the clinical stakeholder and the manufacturing stakeholder (Geethakumari et al., 2021; Gajra et al., 2022). The logistics stakeholder and clinical stakeholders also share the challenge of capacity bottlenecks at hospitals due to a lack of temporary storage for the final product (Calmels et al., 2018; Velickovic and Rasko, 2022). Overall, these interfacing challenges converge on a shared goal to minimize the “vein to vein” time. This is the time it takes from the initial blood materials extraction step to the final product infusion step. Recognizing this shared goal for the supply chain can assist stakeholders in collaborating to implement solutions that target shared challenges.

Singular challenges may also have opportunities for stakeholder collaboration. Toxicity management and patient deterioration are generally considered challenges under the purview of the medical center. Both involve other stakeholders outside of the medical center. The risk of toxic events may be managed to an extent by the medical center, but ultimately is dependent on how the CAR T-cell therapies are designed and changed by the primary manufacturer. The quality of patient cells directly impacts the ability of the manufacturing center to produce sufficient and high-quality products. The responsiveness and policies that surround reimbursement from private and public insurance schemes dictate the level of deterioration of the patient, and ultimately the quality of the starting material. Additionally, the incidence of toxic events determines whether the patient is treated in an in-patient or out-patient facility, which then has an impact on reimbursement for the patient. Currently, there is a degree of collaboration between professional accreditation groups, regulatory bodies and medical centers to work towards mitigating toxicity events. However, coordination between the medical center, the manufacturing facility, insurers and certifying bodies such as regulatory authorities and professional groups must jointly coordinate to appropriately address these indirectly shared challenges.

These areas of shared challenges help formulate aligned goals because they represent the interface between stakeholders (Bunn et al., 2002; Fabbe-Costes et al., 2020; Mubarik et al., 2021). The preconditioning step for the patient involves a line of communication between the clinic and the manufacturer, where one stakeholder must inform the other of the timeline for production and when to start preparing the patient (Papathanasiou et al., 2020). Next, the logistics company must coordinate with the manufacturer and clinic to transport the starting materials and finished cells. The scheduling step is based on the time window where the patient is ready for cell harvesting so that the logistics company can deliver the product before the patient is finished with preconditioning. Often the treatment is delivered well in advance to accommodate changes in patient scheduling, which leads to the capacity bottlenecks at hospitals for storing the frozen product (Lakelin, 2017; Karakostas et al., 2020). Therefore, a shared goal can target communication between stakeholders on the patient conditioning steps, which can reduce waiting times and improve performance measures for the entire supply chain.

Solutions that the literature presents for the cell therapy supply chain tend to either focus on the goals of individual stakeholders or ignore the constraints of inherent supply chain barriers. For example, one of the most highly cited solutions for volumetric scaling of CAR T-cell therapy is decentralized manufacturing (Calmels et al., 2018; Harrison et al., 2019; Lopes et al., 2020; Ortiz de Landazuri et al., 2020; Ran et al., 2020; Triantafyllou et al., 2022; Palani et al., 2023). Decentralized manufacturing has many important benefits such as reducing logistics burdens on the supply chain and patients, while advances in isolated bioreactors like the CliniMACs Prodigy have addressed the specialized infrastructure required (Ran et al., 2020; Palani et al., 2023). However, the strict regulatory requirements for manufacturing make implementation prohibitively expensive, particularly at the lower volumes and unpredictable rates of demand for smaller target populations (Branke et al., 2016; Calmels et al., 2018; Harrison et al., 2019; Borgert, 2021; Myles and Church, 2022). Thus, decentralized manufacturing requires significant investment and involvement from primary, secondary and supporting stakeholders to implement.

Solutions from the cell therapy supply chain literature that involve at minimum all primary stakeholders to overcome the central challenges of the industry can be utilized to implement decentralized manufacturing. As shown in Table 3, there are four solutions that consider the three primary supply chain barriers and require involvement from all three primary stakeholders. This includes a focus on coordination between supply chain stakeholders, improved demand prediction through information integration, cloud-based information sharing and track and trace technology.

**TABLE 3:** Proposed solutions for the CAR T-Cell therapy supply chain.

	Stakeholder involvement			Barriers considered		
Solution	Clinical	Logistics	Manufacturing	Product	Regulatory	Infrastructure
Decentralised manufacturing	X	X	X	X	X	X
Coordination between supply chain stakeholders	X	X	X	X	X	X
Improved demand prediction through improved information integration	X	X	X	X	X	X
Cloud based information sharing	X	X	X	X	X	X
Track and trace technology to maintain chain of custody	X	X	X	X	X	X
Blood and vaccine SC as examples for solutions	X	X	X	X		X
Dynamic supply chain that changes based on demand fluctuations	X	X	X	X		X
Cryopreserved transportation of cells and product	X	X	X	X	X	
Allogenic cell banks	X	X	X			X
Mobile medical units for administration	X	X		X		X
Outpatient treatment to minimise costs and ease reimbursement	X	X		X		X
Automation of quality control processes			X	X	X	X
Outsourcing quality control and quality assurance			X	X	X	X
Automation in manufacturing			X	X		X
Intermediate storage to debottleneck production			X			X
Competition to bring down materials costs			X	X		
Small batch size with isolated product streams			X	X		

Information sharing through track and trace technology addresses concerns on the maintenance of chain of custody and information integration can address the variable demand of lower-volume segments of the population. Stakeholder coordination ensures that the clinical, logistics and manufacturing stakeholders align their individual goals with the overall goals of the supply chain. Overall, this collaborative approach can lower the financial and time investment that a singular stakeholder would need to take on to implement a decentralized manufacturing structure. Additionally, stakeholder collaboration can facilitate the implementation of other key solutions while reducing the burden of risk, cost and adoption time for singular stakeholders. For example, a collaborative approach to addressing toxicity management that shares both risk and financial incentives between stakeholders may allow for the more widespread establishment of certified CAR T-cell medical centers.

Stakeholder coordination as a solution allows supply chain stakeholders to utilize existing resources, align goals, and build a supply chain that meets the needs of the stakeholders through collaboration (Davies and Rafiq, 2017; Myles and Church, 2022). This built-for-purpose supply chain may feature decentralized manufacturing, which will require a collaborative approach among the involved stakeholders to implement solutions that target aligned goals, and overall provide flexibility for stakeholder needs.

## 5 Conclusion

This review presents the challenges of the cell therapy supply chain by considering the primary stakeholders affected and the type of challenge, whether related to product characteristics, regulatory restrictions, or existing infrastructure. Some shared goals including quality tracking and minimized “vein-to-vein” time were identified based on an analysis of the shared challenges of the supply chain. Proposed solutions from the cell therapy literature were evaluated to determine whether they utilize the competencies of the primary stakeholders involved and whether the solutions consider the effects of the core barriers of the supply chain.

The interactions between stakeholders in the supply chain represent the boundaries between supply chain members. These boundaries provide insight into where stakeholder collaboration can be implemented to improve supply chain coordination. Through collaboration, shared challenges translate from aligned goals to an implementation plan to resolve the challenges and meet the goals. As such, there is potential value in moving away from the search for a one-size-fits-all supply chain for cell therapies and toward stakeholder collaboration.

There is an opportunity to convert individual solutions to challenges as opportunities for value creation across the supply chain through stakeholder collaboration. For example, the common regulatory requirement for chain of custody tracking is a shared data collection challenge that involves all stakeholders. Since cell quality tracking and reduced vein-to-vein time is also a shared goal between stakeholders, there is an opportunity to leverage the chain of custody requirement to collect data that can be used to optimize cell quality and reduce waiting times for patients through joint information sharing.

Advances in the cell therapy safety profile will make solutions like outpatient treatment more feasible in the future. Regulatory approvals for allogenic therapy will greatly impact infrastructure development and support centralized manufacturing. Additionally, new therapeutics in cell and gene therapy that are in clinical trials will affect patient eligibility, safety, and manufacturing by expanding the competition for products. This can drive down the cost of raw materials and lower the price of the therapy for patients. In the meantime, it is imperative to encourage collaboration among the stakeholders of the supply chain to implement solutions that target aligned goals and improve supply chain coordination.
